# STK38 Kinase Promotes Cell Migration Induced by Oncogenic Ras via MerTK Activation

**DOI:** 10.3390/ijms262110388

**Published:** 2025-10-25

**Authors:** Satoshi Ohta, Kenji Tago, Katsumi Kasashima, Masayuki Ebina, Kaoru Tominaga

**Affiliations:** 1 Division of Structural Biochemistry, Department of Biochemistry, Jichi Medical University, Shimotsuke 329-0498, Tochigi, Japan; kasa@jichi.ac.jp (K.K.); ebina.masayuki@jichi.ac.jp (M.E.); 2 Department of Laboratory Sciences, Gunma University Graduate School of Health Sciences, 3-39-22 Showa-Machi, Maebashi 371-8514, Gunma, Japan; ktago@gunma-u.ac.jp

**Keywords:** STK38, Ras, MerTK, cell migration

## Abstract

Ras gene mutations are frequently observed in many types of cancers. However, there are currently no effective anticancer drugs against Ras-induced cancers. Therefore, identifying the downstream effectors of the Ras signaling pathway can facilitate the development of promising novel therapeutic approaches. We previously showed that oncogenic Ras induces the expression of the receptor tyrosine kinase c-Mer proto-oncogene tyrosine kinase (MerTK) in an interleukin-1 family member NF-HEV/IL-33-dependent manner and that IL-33 and MerTK contribute to oncogenic Ras-induced cell migration. In the present study, we purified the MerTK complex from NIH-3T3 cells transformed by the expression of oncogenic Ras, H-Ras (G12V). Mass spectrometric analysis identified STK38 (also known as NDR1) as a candidate binding partner for MerTK. STK38 is a serine/threonine protein kinase that plays diverse roles in normal and cancerous cells. In addition to MerTK knockdown, STK38 knockdown effectively attenuated the H-Ras (G12V)-induced migration of NIH-3T3 cells. STK38 kinase activity is required for oncogenic Ras-induced cell migration and MerTK tyrosine phosphorylation. Furthermore, MerTK or STK38 knockdown attenuated the activation of Rac1 and Cdc42. Taken together, these results revealed a novel role for STK38 in oncogenic Ras-induced enhanced cell migration, which may be useful for developing novel therapeutic strategies targeting Ras-mutated cells.

## 1. Introduction

Ras is the most frequently mutated gene family among oncogenes. Within the Ras family, three isoforms of the RAS oncogene, *H-RAS*, *K-RAS*, and *N-RAS*, play important roles in various biological processes, including cell proliferation, differentiation, survival, and migration, in various mammalian cells [1]. The Ras pathway, which includes the Raf/MEK/ERK and PI3K/AKT pathways, is one of the most dysregulated pathways in human cancer [2,3]. Ras proteins are capable of binding to guanine nucleotides, and their activity is controlled by the bound guanine nucleotides; for example, the GDP-bound form is inactive, and the GTP-bound form is active [1,4]. Activating mutations in the Ras gene are associated with multiple cancer types and result in the constitutive activation of the Ras signaling pathway. Although many drugs targeting the Ras signaling pathway have been developed, these targeted therapies do not effectively cure Ras-mutated malignancies [5], suggesting that the mechanism underlying Ras-induced tumorigenesis remains unknown. Therefore, the discovery of new signaling molecules involved in Ras-induced tumor signaling is essential for the development of novel therapeutic strategies.

MerTK is a member of the TAM (Tyro3, Axl, and MerTK) family of receptor tyrosine kinase (RTKs) [6]. MerTK is highly expressed in several types of cancer, such as non–small-cell lung cancer (NSCLC) [7], breast cancer [8,9], colorectal cancer [10,11], glioblastoma multiforme (GBM) [12,13], melanoma [14], acute myeloid leukemia (AML) [15], and schwannoma [16]. MerTK activation triggers multiple pro-oncogenic signaling pathways, including MAPK, p38, and PI3K, which consequently make tumor cells more aggressive in terms of survival, proliferation, and resistance to apoptosis [17]. The vitamin K-dependent proteins Gas6 and Protein S are the most studied ligands of TAM receptors [18,19]. Consistent with other RTKs, ligand binding activates MerTK, followed by the dimerization and autophosphorylation of the kinase domain of MerTK to activate intracellular signaling cascades and regulate gene expression. The number of studies describing the role of MerTK in cancer progression and development, as well as its role in therapeutic strategies, has increased significantly [20].

Previously, we reported that the oncogenic Ras mutant Ras (G12V) enhances the expression of the precursor of IL-33, a nuclear factor or cytokine, and that this Ras/IL-33 pathway is required for Ras-induced cell transformation [21]. Recently, we showed that the Ras/IL-33 pathway promotes cell migration by enhancing MerTK expression and tyrosine phosphorylation [22]. Notably, the cell migration-promoting effect of MerTK was dependent on its autophosphorylation of tyrosine residues but did not require stimulation with a MerTK ligand. The molecular mechanism underlying MerTK activation in the oncogenic Ras signaling pathway remains to be elucidated.

Serine/threonine kinase 38 (STK38, also known as NDR1) is important in a wide variety of biological processes such as cell cycle progression, apoptosis [23], centrosome duplication [24], and cancer biology [25]. STK38 has been shown to have oncogenic potential in progressive ductal carcinoma in situ in breast tissue [26], lung adenocarcinoma [27,28], and ovarian cancer [29]. Therefore, STK38 inhibition has also been considered a novel strategy to target Ras-transformed cells [30].

In the present study, we identified STK38 as a novel MerTK binding protein. We investigated the role of STK38 in the oncogenic Ras signaling pathway in mouse NIH-3T3 cells. By the knockdown of STK38 as well as that of MerTK, cell migration induced by oncogenic Ras was suppressed. More specifically, the kinase activity of STK38 contributed to the tyrosine phosphorylation of MerTK and oncogenic Ras-induced cell migration. In addition, we found that MerTK and STK38 knockdown downregulated the levels of active Rac1 and Cdc42. These results suggest that STK38 regulates oncogenic Ras-induced cell migration through the activation of MerTK and the subsequent activation of Rac1 and Cdc42.

## 2. Results

### 2.1. STK38 Interacts with MerTK

**Figure 1:**
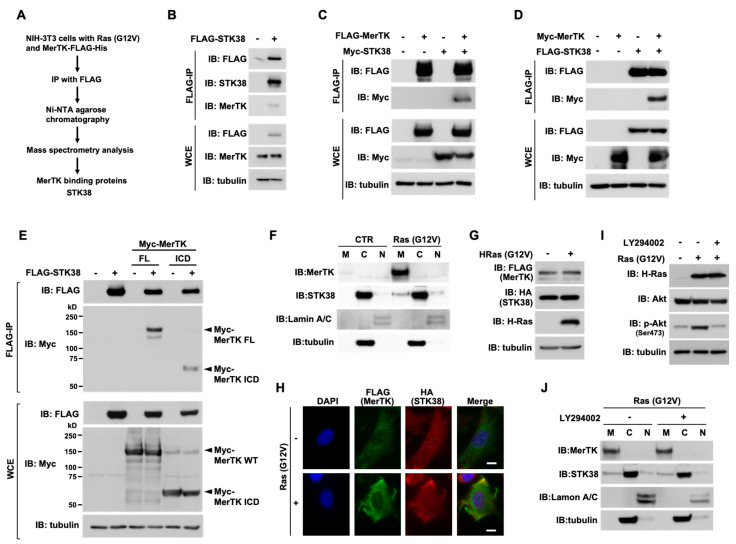
MerTK interacts with STK38. (**A**) The method of purifying MerTK-containing protein complexes using the tandem affinity purification tag technique is schematized. (**B**) HEK293T cells were transfected with expression plasmids for FLAG-STK38 (+) or control (−). Whole cell extracts (WCE) were subjected to immunoprecipitation with anti-FLAG M2 beads. Both WCE and immunoprecipitants (FLAG-IP) were subjected to immunoblot (IB) analysis with an anti-FLAG, anti-STK38, anti-MerTK, and anti-α-tubulin antibodies. Tubulin level was shown as internal loading control. (**C**) HEK293T cells were cotransfected with FLAG-MerTK and Myc-STK38 for 48 h. FLAG-MerTK in WCE was immunoprecipitated with anti-FLAG M2 beads. Immunoprecipitants (FLAG-IP) were analyzed by immunoblotting (IB) with anti-FLAG or anti-Myc antibodies to detect the interacting molecules. Tubulin served as an internal loading control. (**D**) HEK293T cells were cotransfected with FLAG-STK38 and Myc-MerTK for 48 h. FLAG-MerTK in whole cell extracts (WCE) was immunoprecipitated and immunoprecipitants (FLAG-IP) were immunoblotted (IB) as in (**C**). (**E**) HEK293T cells were cotransfected with FLAG-STK38, Myc-tagged full length MerTK (FL), or intracellular domain of MerTK (ICD) for 48 h. FLAG-STK38 in WCE were immunoprecipitated with anti-FLAG M2 beads. Immunoprecipitants (FLAG-IP) were analyzed by immunoblotting (IB) as in (**C**). (**F**) Membrane (M), cytoplasmic (C), and nuclear (N) fractions were prepared from control NIH-3T3 cells (CTR) and NIH-3T3 cells expressing Ras (G12V). Each fraction was analyzed by immunoblotting (IB) with an anti-MerTK, anti-STK38, anti-α-tubulin, and anti-Lamin A/C, antibodies. Tubulin and lamin A/C are detected as a maker for the cytoplasmic and nuclear fractions, respectively. (**G**) NIH-3T3 cells were infected with the indicated combinations of retroviral constructs encoding Ras (G12V) (+), FLAG-MerTK, HA-STK38. Whole cell extracts were analyzed by immunoblotting (IB) with an anti-FLAG, anti-HA, anti-H-Ras, and anti-α-tubulin antibodies. Tubulin served as an internal loading control. (**H**) NIH-3T3 cells were infected with the same combination of the retroviral constructs in (**G**) were subjected to the immunofluorescence analysis. The localization of MerTK and STK38 were detected using anti-FLAG and anti-HA antibodies, respectively. Nuclei were visualized by DAPI. The scale bar represents 10 μm. (**I**) NIH-3T3 cells infected with retroviral constructs encoding Ras (G12V) (+) or an empty vector (−) were treated with 50 μM of LY294002 for 24 h. Whole cell extracts were analyzed by immunoblotting (IB) with an anti-H-Ras, anti-Akt, anti-phospho-Akt (Ser473), and anti-α-tubulin antibodies. Tubulin was used as internal loading control. (**J**) NIH-3T3 cells expressing Ras (G12V) were treated with 50 μM of LY294002 for 24 h. Cells were fractionated and each fraction was analyzed by immunoblotting (IB) as in (**F**).

We previously demonstrated that oncogenic Ras induces the expression of IL-33 and Ras/IL-33 signaling pathway induces the expression of MerTK, which contributes to the cell migration enhanced by oncogenic Ras in MerTK ligands-independent manner [22]. To identify potential regulators that contribute to Ras/IL-33/MerTK pathway, we performed a tandem affinity purification to identify a novel component of MerTK complex. We expressed FLAG-His-tagged MerTK concomitant with oncogenic Ras, Ras (G12V), in NIH-3T3 cells and purified the MerTK-containing protein complexes using immunoprecipitation with the anti-FLAG M2 beads followed by nickel agarose chromatography (Figure 1A). By using mass spectrometry, we identified nine proteins including serine/threonine kinase STK38 as a novel MerTK binding protein, which we regarded as a potential regulator of Ras/IL-33/MerTK signaling pathway (Supplementary Table S1). To verify whether STK38 harbors the binding ability to MerTK, FLAG-tagged STK38 was transiently overexpressed in HEK293T cells, immunoprecipitated with anti-FLAG M2 beads, and probed with antibodies against FLAG, STK38, and MerTK. As shown in Figure 1B, we found that endogenous MerTK coimmunoprecipitated with STK38. We also performed immunoprecipitation using lysates prepared from cells expressing FLAG-MerTK and Myc-STK38 or FLAG-STK38 and Myc-MerTK. FLAG-MerTK and FLAG-STK38 were immunoprecipitated with anti-FLAG M2 beads and immunoblotted with antibodies against FLAG or Myc. In each experiment, co-precipitation of STK38 or MerTK was observed (Figure 1C,D). To investigate whether STK38 interacts with the intracellular domain of MerTK, we performed immunoprecipitation using cell lysates prepared from cells expressing FLAG-STK38 and full-length MerTK (named MerTK FL) or the intracellular domain of MerTK (named MerTK-ICD). Same as MerTK FL, MerTK ICD also co-precipitated with STK38 (Figure 1E). While MerTK is a transmembrane receptor tyrosine kinase [6], STK38 is reported to be mostly localized in the cytoplasm [31]; therefore, to explore the intracellular localization of MerTK and STK38, we biochemically fractionated lysates prepared from Ras (G12V) expressing cells into membrane, cytosolic, and nuclear fractions, followed by immunoblotting (Figure 1F). As in our previous report [22], Ras (G12V) strongly induced the expression of MerTK, which was mainly detected in the cell membrane fraction. Consistent with previous reports [31], STK38 was detected in the cytoplasmic fraction in Ras (G12V) no-expressing cells. On the other hand, in Ras (G12V) expressing cells, STK38 was detected in the membrane fraction to some extent. We also observed that MerTK and STK38 co-localized near the plasma membrane in cells expressing Ras (G12V) by performing immunostaining (Figure 1G,H). To determine whether PI3K/Akt pathway, a typical Ras effector pathway [32], plays a role in the membrane translocation of STK38, cells were treated with an inhibitor of PI3K, LY294002. In cells expressing Ras (G12V), LY294002 inhibited the phosphorylation of Akt (Ser473) (Figure 1I), but had no effect on the translocation of STK38 to the membrane (Figure 1J). These results suggested that a part of STK38 is translocated to the plasma membrane independently of PI3K/Akt pathway and interacts with MerTK in Ras (G12V) expressing cells.

### 2.2. STK38 Contributes to Ras (G12V)-Induced Cell Migration

**Figure 2:**
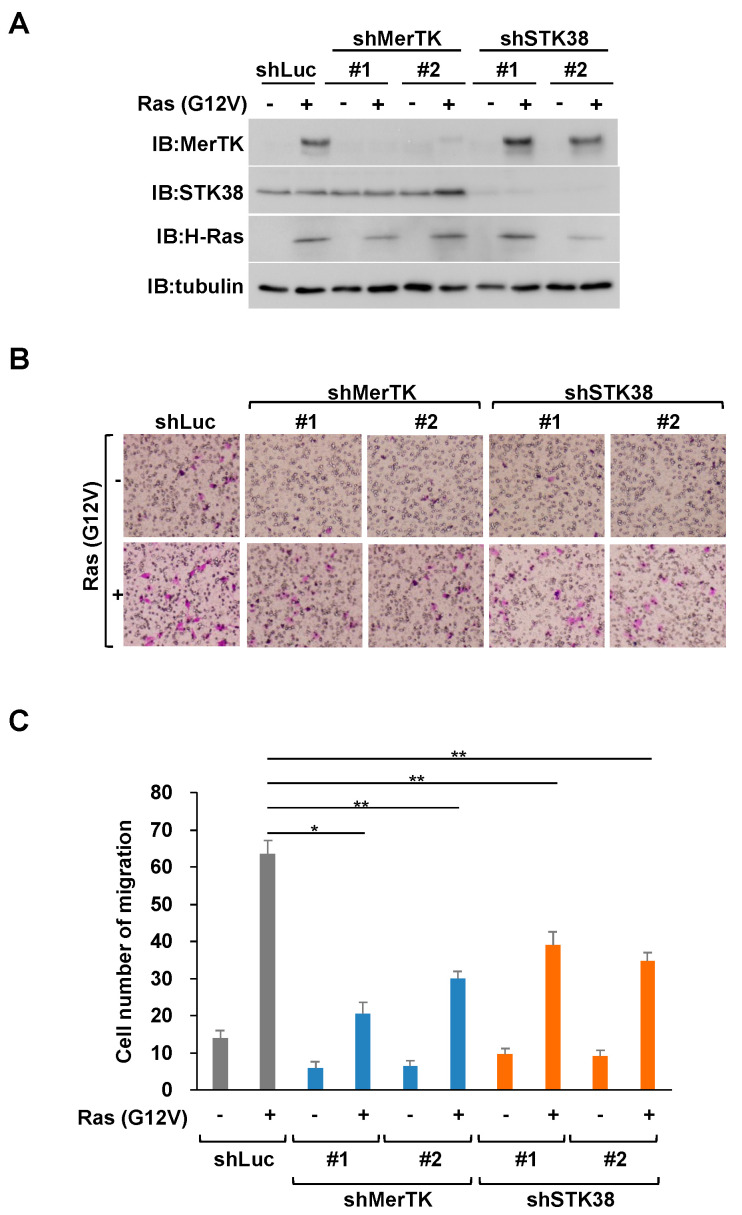
Knockdown of STK38 suppresses cell migration induced by Ras (G12V). (**A**) NIH-3T3 cells were infected with various combinations of retroviral constructs encoding MerTK shRNA (shMerTK #1 or #2), STK38 shRNA (shSTK38 #1 or #2), Ras (G12V) (+), an empty vector (−), or control luciferase shRNA (shLuc). Whole cell extracts were subjected to immunoblotting (IB) with an anti-MerTK, anti-STK38, anti-H-Ras, and anti-α-tubulin antibodies. Tubulin served as an internal loading control. (**B**,**C**) NIH-3T3 cells infected with the same combination of the retroviral constructs in (**A**) were analyzed by transwell migration assay. Migrated cells were observed with 4.2× objective lens (FSX100, Olympus, Tokyo, Japan). Four fields of cells in the lower side were counted, and the counted cell number was shown as a graph. Error bars represent the mean ± SD of three independent biological replicates. *, and ** indicate *p* < 0.05 and *p* < 0.01, respectively.

**Figure 3:**
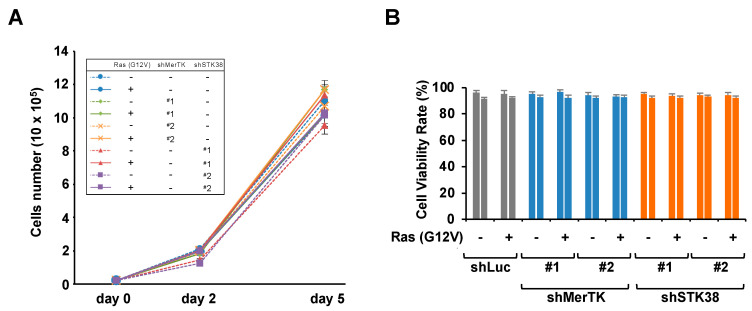
Knockdown of STK38 does not affect the cell growth and cell viability in NIH-3T3 cells. (**A**) Growth curve of NIH-3T3 cells infected with the same combination of the retrovirus constructs in Figure 2A. The cell number was counted at day 0, 2, and 5. (**B**) NIH-3T3 cells were infected with the same combination of the retrovirus constructs in Figure 2A and cell viability was investigated by trypan blue exclusion. Error bars represent the mean ± SD of three independent biological replicates.

To clarify whether STK38 is involved in the enhancement of cell migration induced by Ras-MerTK signaling, we investigated the effect of STK38 on cell migration induced by oncogenic Ras. Using retroviral gene transfer techniques, we initially prepared the following cells: NIH-3T3 cells transformed by H-Ras (G12V) expression (named NIH-3T3^RasV^ cells) and NIH-3T3^RasV^ cells in which MerTK or STK38 expression were knocked down by shRNA (named NIH-3T3^RasV/shMerTK^ or NIH-3T3^RasV/shSTK38^ cells) (Figure 2A). These cells were then subjected to in vitro migration assays using transwell chambers (Figure 2B,C). The number of NIH-3T3^RasV^ cells that had migrated through the membrane was almost fourfold higher than the number of control cells. Consistent with our previous study [22], MerTK knockdown attenuated Ras (G12V)-induced cell migration. Similarly, we found that STK38 knockdown significantly attenuated Ras (G12V)-induced cell migration, suggesting an indispensable role for STK38 in Ras (G12V)-induced cell migration. We examined whether STK38 knockdown affected the proliferation and viability of NIH-3T3 cells (Figure 3A,B). Consistent with our previous study [22], MerTK knockdown did not affect the proliferation or viability of NIH-3T3 cells. Similarly, STK38 knockdown did not affect them. These results indicated that the attenuation of migration by STK38 knockdown was not because of a decrease in cell proliferation or viability.

### 2.3. STK38 Kinase Activity Is Necessary for STK38-Mediated Enhancement of Ras (G12V)-Induced Cell Migration

**Figure 4:**
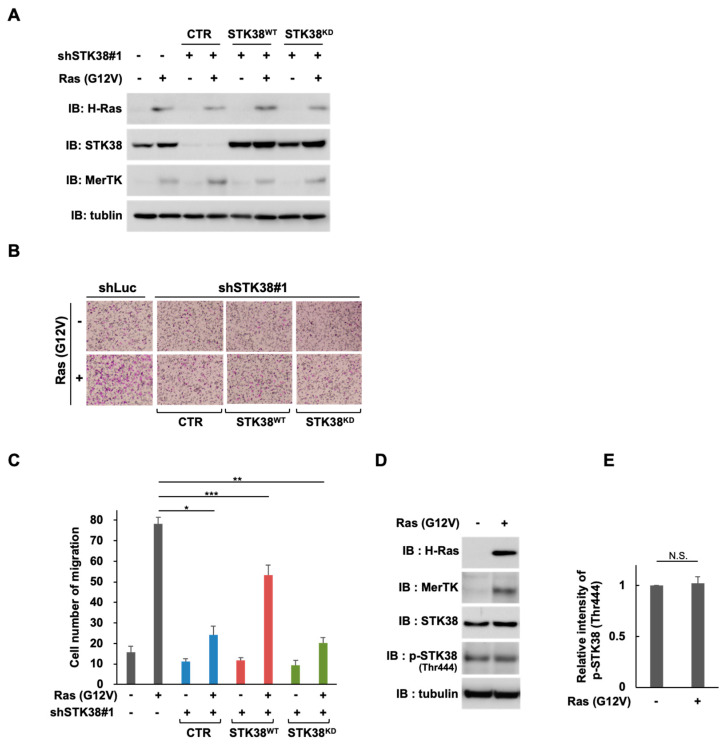
The kinase activity of STK38 is required for oncogenic Ras-induced cell migration. (**A**) NIH-3T3 cells were infected with various combinations of retroviral constructs encoding STK38 shRNA (shSTK38 ^#^1), Ras (G12V), control luciferase shRNA (shLuc), wild-type FLAG-STK38 (STK38^WT^), the FLAG-STK38 kinase-dead mutant (STK38^KD^), and an empty vector (CTR). Whole cell extracts were analyzed by immunoblotting (IB) with an anti-H-Ras, anti-STK38, anti-MerTK, and anti-α-tubulin antibodies. Tubulin served as an internal loading control. (**B**,**C**) NIH-3T3 cells infected with the same combination of the retroviral constructs indicated in (**A**) were subjected to the transwell migration assay. The cells on the lower side in four fields were observed with 4.2× objective lens (FSX100, Olympus) and counted and shown as a graph. Error bars represent the mean ± SD of three independent biological replicates. *, **, and *** indicate *p* < 0.05, *p* < 0.01, and *p* < 0.005, respectively. (**D**) NIH-3T3 cells infected with the combination of retroviral constructs encoding Ras (G12V) (+) and an empty vector (−). Whole cell extracts were analyzed by immunoblotting (IB) with an anti-H-Ras, anti-MerTK, anti-STK38, anti-phospho-STK38 (Thr444), and anti-α-tubulin antibodies. Tubulin was used as internal loading control. (**E**) The intensity of p-STK38 signals in (**D**) were normalized with those of tubulin signals and the relative intensity of p-STK38 signals were shown as a graph. Error bars represent the mean ± SD of three independent biological replicates. N.S., not significant.

Because STK38 is known to be a serine/threonine kinase possessing various roles in the cell cycle and cancer biology [33], we examined whether the kinase activity of STK38 is required for Ras-mediated acceleration of cell migration. We composed a retroviral construct harboring the kinase-dead mutant of STK38 (named STK38^KD^) [34]. Prior to the expression of wild-type STK38 (named STK38^WT^) or STK38^KD^, endogenous STK38 was knocked down by shRNA targeting the 3′ untranslated region of SKT38, which exerted no effect on the exogenous expression of STK38^WT^ or STK38^KD^. As shown in Figure 4A, STK38^WT^ and STK38^KD^ were both expressed in the endogenous STK38-silenced background. The expression of STK38^WT^ promoted cell migration of STK38 knockdown cells (Figure 4B,C). On the other hand, STK38^KD^ failed to accelerate the cell migration, suggesting that the kinase activity of STK38 is indispensable for the migration of Ras-transformed NIH-3T3 cells. To explore whether Ras (G12V) regulates the level of STK38 protein or the activation of STK38, we examined those in NIH-3T3 cells expressing Ras (G12V). As shown in Figure 4D,E, Ras (G12V) did not affect the protein level of STK38 and the phosphorylation of threonine at 444, which indicates the STK38 activation [35].

### 2.4. STK38 Promotes the Tyrosine Phosphorylation of MerTK Depending on Its Kinase Activity

**Figure 5:**
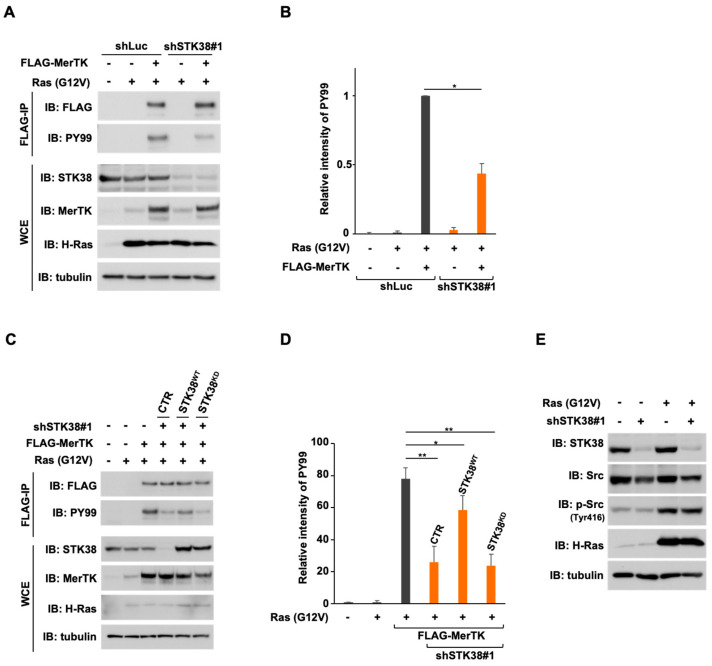
The kinase activity of STK38 promotes the tyrosine phosphorylation of MerTK. (**A**) NIH-3T3 cells were infected with various combinations of retroviral constructs encoding Ras (G12V), STK38 shRNA (shSTK38 ^#^1), control luciferase shRNA (shLuc). Whole cell extracts (WCE) were subjected to immunoprecipitation with anti-FLAG M2 beads. Both WCE and immunoprecipitants (FLAG-IP) were subjected to immunoblot analysis with an anti-FLAG, anti-phosphotyrosine (PY99), anti-STK38, anti-MerTK, anti-H-Ras, and anti-α-tubulin antibodies. Tubulin served as an internal loading control. (**B**,**D**) The intensity of PY99 signals in (**A**) or (**C**) were normalized with those of FLAG signals and the relative intensity of PY99 signals were shown as a graph in (**B**) or (**D**), respectively. Error bars represent the mean ± SD of three independent biological replicates. *, and ** indicate *p* < 0.05 and *p* < 0.01, respectively. (**C**) NIH-3T3 cells were infected with various combinations of retroviral constructs encoding Ras (G12V), STK38 shRNA (shSTK38 ^#^1), luciferase shRNA (shLuc) as a control shRNA, FLAG-MerTK, wild-type FLAG-STK38 (STK38^WT^), FLAG-STK38 kinase-dead mutant (STK38^KD^), and an empty vector (CTR). Whole cell extracts (WCE) were subjected to immunoprecipitation with anti-FLAG M2 beads. Both WCE and immunoprecipitants (FLAG-IP) were subjected to immunoblot (IB) analysis with an anti-FLAG, anti-phosphotyrosine (PY99), anti-STK38, anti-MerTK, anti-H-Ras, and anti-α-tubulin antibodies. Tubulin was used as internal loading control. (**E**) NIH-3T3 cells were infected with the combination of retroviral constructs encoding Ras (G12V), STK38 shRNA (shSTK38 ^#^1), luciferase shRNA as a control shRNA. Whole cell extracts were subjected to immunoblot analysis with an anti-STK38, anti-Src, anti-phospho-Src (Tyr416), anti-H-Ras, and anti-α-tubulin antibodies. Tubulin was used as internal loading control.

To gain further insight into the mechanism by which the physical interaction between MerTK and STK38 contributes to cell migration, we prepared FLAG-tagged MerTK in NIH-3T3^RasV/shSTK38^ cells (named NIH-3T3^RasV/shSTK38/F-MerTK^ cells). After lysis of the cells, FLAG-tagged MerTK were precipitated with anti-FLAG M2 beads and probed with anti-phosphotyrosine antibody (PY99) to detect tyrosine phosphorylation on MerTK, which indicates the MerTK activation (Figure 5A,B). The knockdown of STK38 efficiently attenuated the tyrosine phosphorylation of MerTK, suggesting that STK38 promotes the activation of MerTK. To investigate whether STK38 kinase activity plays a role in supporting MerTK tyrosine phosphorylation, we prepared wild-type STK38 or kinase-dead STK38 expressing NIH-3T3^RasV/shSTK38/F-MerTK^ cells and detected the tyrosine phosphorylation of MerTK (Figure 5C,D). In this experiment, kinase-dead STK38 was expressed at approximately equivalent levels of wild-type STK38 (Figure 5C). The suppression of the tyrosine phosphorylation on MerTK by the knockdown of STK38 was partially restored by the expression of wild-type STK38, but not by the expression of kinase-dead STK38. c-Src is a non-receptor tyrosine kinase, a member of the Src kinase family (SFK) [36], that has been reported to mutually activate Ras and promote tumorigenesis [37,38]. MerTK signaling has been reported to crosstalk with αvβ5 integrins and activate focal adhesion kinase (FAK) via the SFK [39]. It has also been reported that the intracellular domain of MerTK interacts with Src [40], resulting in enhanced phosphorylation of Src at Tyr416 [41], which indicates activation of Src. To investigate the potential effect of STK38 on the activation of Src, we subsequently examined whether knockdown of STK38 affects the activities of Src. The phosphorylation of Src at Tyr416 was enhanced by expression of Ras (G12V), but this enhancement was not affected by STK38 knockdown (Figure 5E). These findings suggested that the kinase activity of STK38 contributes to support the tyrosine phosphorylation of MerTK in Src-independent manner.

### 2.5. MerTK and STK38 Promote Rac1 and Cdc42 Activation

**Figure 6:**
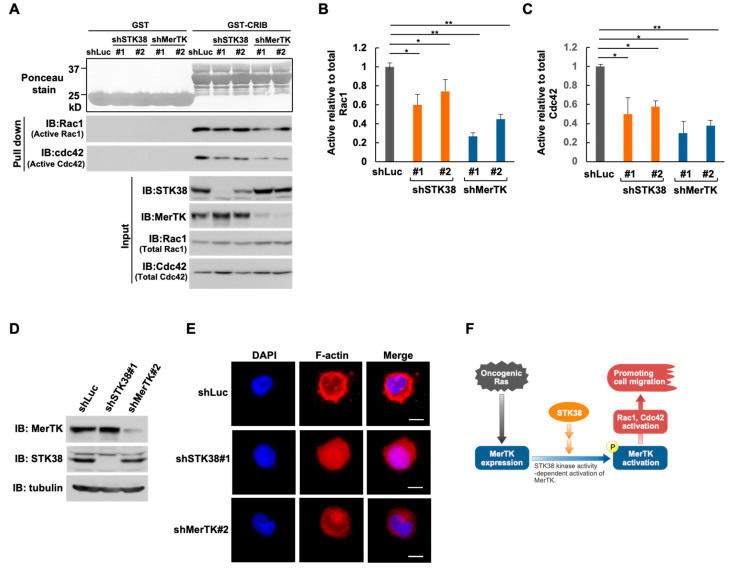
Knockdown of MerTK and STK38 reduce active Rac1 and Cdc42. (**A**) HEK293T cells were infected with various combinations of retroviral constructs encoding STK38 shRNA (shSTK38 #1 and #2), MerTK shRNA (shMerTK #1 and #2), and control Luciferase shRNA (shLuc). Whole cell extracts were subjected to pulldown with GST-CRIB or GST to collect active Rac1 and Cdc42. The amount of Rac1 and Cdc42 in the pulldown assay was determined by immunoblotting (IB) with anti-Rac1 and anti-Cdc42 antibodies. Whole cell extracts (Input) were analyzed by immunoblotting (IB) with an anti-STK38, anti-MerTK, anti-Rac1, and anti-Cdc42 antibodies. Ponceau red stain was used to indicate the GST-fused protein amount. (**B**,**C**) The ratios of active Rac1 (**B**) and active Cdc42 (**C**) to total Rac1 and Cdc42 are shown in the graph. Data obtained from three independent experiments are expressed as the mean SD. *, and ** indicate *p* < 0.05 and *p* < 0.01, respectively. (**D**) HEK293T cells were infected with various combinations of retroviral constructs encoding STK38 shRNA (shSTK38 #1), MerTK shRNA (shMerTK #2), control luciferase shRNA (shLuc). Whole cell extracts were subjected to immunoblot analysis with an anti-STK38, anti-MerTK, and anti-α-tubulin antibodies. Tubulin served as an internal loading control. (**E**) HEK29T cells were infected with the same combination of the retroviral constructs in (**D**) were subjected to the immunofluorescence analysis. Cells were fixed and stained with phalloidin (red) to detect F-actin. Nuclei were visualized by DAPI. The scale bar represents 10 μm. (**F**) Schematic illustration of the role of STK38 in oncogenic Ras-induced cell migration.

The Rho family GTPase Rac1 and Cdc42 play important roles in cytoskeleton rearrangement [42]. It has also been reported that MerTK accelerated the phosphorylation of Vav1, a guanine nucleotide-exchange factor (GEF), which activates Rac1, Cdc42, and RhoA, and the knockdown of MerTK expression reduced cell migration in a Cdc42-dependent manner [43]. These findings let us hypothesize that STK38, as well as MerTK, also function as an activator of Rho family members in oncogenic Ras signaling pathway. To verify this hypothesis, we investigated whether knockdown of MerTK and STK38 affect the activities of Rac1 and Cdc42. The levels of active GTP-bound Rac1 and Cdc42 in HEK293T cells with MerTK or STK38 knockdown were determined by a specific pulldown assay using the Cdc42/Rac1 interacting binding (CRIB) domain of Pak1 (Figure 6A–C). In cells with MerTK or STK38 knockdown, both active Rac1 and Cdc42 were significantly reduced, suggesting that both MerTK and STK38 contribute the activation of Rac1 and Cdc42. Consistent with the results of the pulldown assay, knockdown of MerTK and STK38 caused a striking inhibition of both lamellipodia and filopodia formation, as compared to control, shLuc-treated cells (Figure 6D,E).

## 3. Discussion

We have reported that expression of the receptor tyrosine kinase MerTK is upregulated by oncogenic Ras mutants, and that MerTK contributes Ras-enhanced cell migration [22]. In this study, we identified STK38 as a novel MerTK-binding protein and hypothesized that STK38 may be involved in the regulation of Ras-induced cell migration via MerTK. To support this hypothesis, the knockdown of STK38 suppressed cell migration induced by Ras (G12V). In addition, the kinase activity of STK38 contributed to enhanced cell migration induced by Ras (G12V) and the tyrosine phosphorylation of MerTK. Furthermore, STK38 and MerTK were required for the activation of Rac1 and Cdc42. Overall, our studies demonstrate a key role for STK38 in regulating oncogenic Ras-induced cell migration by promoting the activation of MerTK. The role of STK38 in oncogenic Ras-induced cell migration predicted from this study is summarized in Figure 6F.

STK38 has been shown to be required for the egress of mature thymocytes from the thymus and the interstitial migration of naive T cells [44]. It has also been reported that the knockdown of STK38 inhibits anoikis resistance, anchorage-independent soft agar growth, and in vivo xenograft growth of Ras-transformed human cells [30], shedding light on the supporting oncogenic role of STK38 in cell migration in Ras-dependent cancer cells. In this context, the STK38 knockdown suppressed Ras (G12V)-induced cell migration in NIH-3T3 cells (Figure 2B,C). In addition, we found that only wild-type STK38, but not kinase-dead STK38, restored the reduction in Ras (G12V)-induced cell migration caused by STK38 knockdown (Figure 4B,C). These findings suggest that the kinase activity of STK38 contributes to the oncogenic Ras-induced cell migration and subsequently to tumor metastasis.

We previously reported that tyrosine phosphorylation of MerTK is induced by Ras-induced signals in a ligand-independent manner, suggesting that MerTK activation occurs through intracellular signaling [22]. In the present study, we found that knockdown of STK38 attenuated the tyrosine phosphorylation of MerTK (Figure 5A,B), suggesting that STK38 contributes to ligand-independent activation of MerTK. STK38 is known to be involved in G1/S cell cycle progression through regulating MYC and p21 protein levels. STK38 directly binds to and increases in MYC levels, a transcription factor that regulates cell proliferation, growth, apoptosis, and differentiation, independent of its kinase activity [45]. On the other hand, STK38 phosphorylates p21 at Ser146, inducing its degradation and regulating G1/S cell cycle progression [23]. Several other substrates of STK38 kinase are known, including the G2/M checkpoint protein CDC25A [46], the transcription factor YAP [47], and the nuclear export protein XPO1 [48]. Furthermore, the kinase activity of STK38 has been shown to be required for chromosome alignment during cytokinesis [49] and centrosome duplication [24], although the substrates of STK38 remain unknown. The present results demonstrated that wild-type STK38, but not kinase-dead STK38, restored the reduced tyrosine phosphorylation of MerTK caused by STK38 knockdown (Figure 4C,D), suggesting that the kinase activity of STK38 promotes the tyrosine phosphorylation of MerTK.

We confirmed that the kinase activity of STK38 contributes to the tyrosine phosphorylation of MerTK, which is required for Ras (G12V)-induced cell migration, but how STK38 is involved in the activation of MerTK remains unclear. On the other hand, Ras (G12V) did not affect the stability and the activation of STK38 (Figure 4D,E). We observed that STK38 binds to the intracellular domain of MerTK (Figure 1E), but we have not shown that STK38 directly phosphorylates MerTK. Src tyrosine kinase has also been reported to bind to the intracellular domain of MerTK [40], but knockdown of STK38 does not affect Src activation (Figure 5E), suggesting that Src is not directly involved in the phosphorylation of MerTK. It is well established that MST1 and MST2, the main kinase in the Hippo pathway, phosphorylate STK38 on Thr444, induce the activation of the kinase [50,51]. Additionally, MAP4K kinase, which is a member of the Ste20-like kinases family, regulates STK38 through phosphorylation [52]. In the subcellular fractionation experiment, a part of STK38 was localized in the cell membrane fraction same as MerTK in Ras (G12V)-expressing cells (Figure 1F). It is also known that STK38 activity is constitutively activated when STK38 colocalizes at the plasma membrane with human MOB proteins, which regulates STK38 activation [34]. These findings may suggest that MerTK is activated by crosstalk between Ras signaling pathway and another signaling pathway involving STK38.

Metastasis from primary tumors to other tissues is the main cause of cancer death [53]. The Ras family is frequently mutated in human cancers and regulates cell proliferation, adhesion, and migration [54]. Previous studies have reported that MerTK promotes melanoma cell migration [14,55] and glioma multiforme cell migration, invasion, and survival [12,56]. In this study, we observed that STK38 promoted Ras-induced cell migration (Figure 2) and tyrosine phosphorylation of MerTK (Figure 5A,B), suggesting that STK38 is most likely involved in Ras-induced cell migration and may facilitate tumor metastasis. Ras has also been shown to mediate the activation of Rho family proteins through PI3K [57]. The Rho family play a major role in cell migration and in the metastatic process, and Rac, RhoA, and Cdc42 are representative members that function in cell migration [58]. It has also been reported that MerTK activates the Rho family, and knockdown of MerTK attenuates Cdc42-dependent cell migration [14]. We observed that knockdown of MerTK or STK38 reduced active Rac1 and Cdc42 (Figure 6). These findings suggest that STK38 function as activators of Rac1 and Cdc42 in oncogenic Ras signaling via the tyrosine phosphorylation of MerTK to promote cell migration.

Taken together, our data demonstrate that STK38 promotes tyrosine phosphorylation of MerTK, thereby enhancing Ras-induced cell migration in NIH-3T3 cells. Moreover, STK38 kinase activity contributes to tyrosine phosphorylation of MerTK and Ras-induced cell migration. Although the molecular mechanism by which STK38 contributes to the tyrosine phosphorylation of MerTK is not yet fully understood, our findings provide further understanding and imply a new target for tumor metastasis therapy.

## 4. Materials and Methods

### 4.1. Cell Cultures

Murine NIH-3T3 fibroblasts and human embryonic kidney 293T (HEK293T) cells were cultured in Dulbecco’s modified Eagle’s medium (DMEM) (Nacalai Tesque, Kyoto, Japan) supplemented with 10% fetal bovine serum (FBS) (Merck Millipore, Darmstadt, Germany) and antibiotics (Merck Millipore). Cells were incubated in a humidified atmosphere at 37 °C with 5% CO_2_. The PI3K inhibitor, LY294002, was purchased from FUJIFILM Wako Pure Chemical Corporation (Tokyo, Japan).

### 4.2. Plasmid Constructs

Mouse MerTK, intracellular domain of MerTK (MerTK ICD, amino acids 521-994), and STK38 coding sequence were obtained through PCR amplification from NHI-3T3 cell-derived cDNA and subcloned into p3xFLAG-CMV10 (Merck Millipore, Burlington, MA, USA), pcDNA3-3xFLAG, pcDNA3-Myc (Thermo Fisher Scientific, Waltham, MA, USA), and MSCV-puro-3xFLAG-6xHis vectors. The STK38 Kinase-dead mutant K118A [34] was generated by PrimeSTAR Mutagenesis Basal Kit (Takara Bio Inc., Shiga, Japan) using the following primers: forward 5′-TGTACGCAATGGCAATCCTGCGCAA and reverse 5′-TTGCGCAGGATTGCCATTGCGTACA. The *Escherichia coli* expression plasmid encoding the Cdc42/Rac1 interacting binding (CRIB) domain of Pak1 was constructed and introduced into *E. coli* BL21. GST-CRIB was purified and used for a pulldown assay.

### 4.3. Retroviral Gene Expression

To prepare the retrovirus, HEK293T cells were seeded on a 60 mm dish and transfected with a combination of plasmids, including helper vectors pE-Eco, pGP (Takara Bio Inc.), or PCI-VSVG (gifted by Garry Nolan, Addgene plasmid # 1733), and retroviral backbone plasmids, such as pBabePuro and MSCV-ires-GFP, using FuGENE HD transfection reagent (Promega, Madison, WI, USA). pBabePuro was used to express the G12V H-Ras mutant. MSCV-puro cells were used to express FLAG-tagged MerTK, STK38, or a kinase-dead mutant. After 24 h, the culture medium of the transfected cells was changed with 1.7 mL fresh DMEM containing FBS. After another 12 h, conditioned medium containing retroviruses was collected every 4 h at least 4 times. Exponentially growing cells at 2 × 10^5^ cells in 60 mm culture dish were infected with 1.7 mL of the collected culture supernatant containing retroviruses with 1 μg/mL polybrene (Merck Millipore) for 2 h. Infection efficiencies were confirmed by puromycin selection.

### 4.4. Tandem Affinity Purification of Tagged MerTK Protein and Identification of MerTK-Interacting Proteins

MerTK cDNA was amplified using PCR as previously described [22] and inserted into the MSCV vector containing the C-terminal 6xHis and FLAG tags. NIH-3T3 cells transfected with the vector were lysed in RIPA buffer (25 mM Tris-HCl, pH7.5, 150 mM NaCl, 1% NP-40, 1% sodium deoxycholate), supplemented with a protease inhibitor and phosphatase inhibitor (Nacalai Tesque, Kyoto, Japan). The lysates were cleared by centrifugation at 13,500× *g* for 15 min at 4 °C, and the resulting supernatant was loaded in the anti-FLAG M2 affinity gel (Merck Millipore) and rotated at 4 °C for 2 h. The resin was washed five times with RIPA buffer and bound proteins were eluted using 100 ug/mL FLAG peptide in RIPA buffer. The eluted fractions were next loaded into Ni-NTA agarose (Qiagen, Valencia, CA, USA) and rotated at 4 °C for 2 h. The resin was washed five times with RIPA buffer, and FLAG-tagged MerTK-containing protein complexes were eluted using elution buffer (phosphate-buffered saline [PBS], 250 mM imidazole). FLAG-tagged MerTK-containing protein complexes were separated by sodium dodecyl sulfate-polyacrylamide gel electrophoresis (SDS-PAGE), and each lane was sliced into 20 pieces. Each gel slice was subjected to reduction with 10 mM dithiothreitol (DTT) for 45 min at 56 °C, alkylation with 55 mM iodoacetamide for 30 min at room temperature in a dark place, and tryptic digestion with 20 ug/mL trypsin (Promega, Fitchburg, WI, USA) at 37 °C for 12 h. The tryptic peptides were extracted with 5% trifluoroacetic acid and acetonitrile, concentrated under vacuum, and desalted using Zip-Tip (Merck Millipore). The resulting peptides were mixed with equal volumes of matrix (a saturated solution of α-cyano-4-hydroxy cinnamic acid in 2:1 [*v*/*v*] 0.1% trifluoroacetic acid/acetonitrile) and spotted on a standard stainless steel 384-sample MALDI target plate. MALDI-TOF spectra were obtained using an AutoFlex speed mass spectrometer (Bruker Daltonics, Bremen, Germany) and raw data were analyzed using the Mascot server (Matrix Science, Tokyo, Japan) to identify high-scoring proteins.

### 4.5. Cellular Fractionation

NIH-3T3 cells infected with H-Ras (G12V) retroviruses were harvested, lysed with hypotonic buffer (10 mM HEPES-KOH pH 7.8, 10 mM KCl, 0.1 mM EDTA) supplemented with a protease inhibitor and phosphatase inhibitor (Nacalai Tesque), and then homogenized using a 27G needle. The nuclear pellet was collected by centrifugation at 600× *g* for 2 min at 4 °C. The pellet was resuspended with nuclear lysis buffer (50 mM HEPES-KOH pH 7.8, 420 mM KCl, 0.1 mM EDTA, 5 mM MgCl_2_, 2% glycerol) supplemented with a protease inhibitor and phosphatase inhibitor (Nacalai Tesque) and rotated for 30 min at 4 °C. After being centrifugated at 17,000× *g* for 15 min at 4 °C, the supernatant was collected as the nuclear extract. The supernatant of the first centrifugation at 600× *g* was subsequently ultracentrifuged at 100,000× *g* at 4 °C for 1 h. The supernatant was collected and used as the cytoplasmic extract. The pellet of the ultracentrifugation was suspended in 1% Triton X-100 and incubated for 12 h at 4 °C, and then nuclear lysis buffer was added to adjust to the Triton X-100 concentration 0.1%. This extract constitutes the membrane fraction. Equal amounts of each fraction (cytoplasmic, membrane, and nuclear fractions) were subjected to SDS-PAGE.

### 4.6. Immunoblotting

Cells were washed twice with PBS, and whole cellular extracts were prepared using RIPA buffer supplemented with protease and phosphatase inhibitors (Nacalai Tesque). Lysates were cleared by centrifugation and proteins were separated by SDS-PAGE and transferred to an Immobilon-P ^®^ Transfer Membrane (Merck Millipore). The membranes were blocked in PBS supplemented 0.1% Tween 20 and 5% (*w*/*v*) non-fat dry milk. The membranes were proved with anti-α-tubulin (Merck Millipore, T6199), anti-MerTK (R&D systems [Minneapolis, MN, USA], AF591), anti-STK38 (Abnova corporation [Taipei, Taiwan], 00011329-MO2), anti-phosho-STK38 (Thermo Fisher Scientific, PA5-99570), anti-H-Ras (Santa Cruz Biotechnology [Santa Cruz, CA, USA], sc-35), anti-phosphotyrosine (PY99) (Santa Cruz Biotechnology), anti-Rac1 (BD Transduction Laboratories [Franklin Lakes, NJ, USA], 610650), anti-Cdc42 (Cell Signaling Technology [Danvers, MA, USA], 2462), anti-Lamin A/C (Santa Cruz Biotechnology, sc-6215), anti-FLAG (Merck Millipore, F3165), anti-Myc (proteintech [Rocky Hill, NJ, USA], 16286-1-AP), anti-Akt (Cell Signaling Technology, 4691), anti-phospho-Akt (Cell Signaling Technology, 4060), anti-Src (Santa Cruz Biotechnology, sc-130124), anti-phospho-Src (Cell Signaling Technology, 6943) antibodies. Horseradish peroxidase (HRP)-conjugated goat anti-mouse (Cytiva [Marlborough, MA, USA], NA931V), goat anti-rabbit (Cytiva, NA934V), and bovine anti-goat (Santa Cruz Biotechnology, sc-2378) secondary antibodies were used. The immunoreactive bands were visualized using enhanced chemiluminescence (Nacalai Tesque).

### 4.7. Immunoprecipitation

HEK293T cells were transfected with plasmid DNA using FuGENE HD (Promega) and incubated for 48 h. Cell lysates were prepared using RIPA buffer supplemented with protease inhibitor (Nacalai Tesque) and phosphatase inhibitor (Nacalai Tesque). For immunoprecipitation, cell lysates were incubated with anti-FLAG M2 beads (Merck Millipore) at 4 °C for 2 h, and beads were then washed with RIPA buffer three times. Proteins were eluted in Laemmli sample buffer by boiling. To validate the tyrosine phosphorylation levels of MerTK, NIH-3T3 cells were infected with retroviruses expressing Ras (G12V), FLAG-MerTK, shSTK38, wild-type STK38, or the kinase-dead mutants. Cells were lysed in RIPA buffer, and immunoprecipitation was performed using anti-FLAG M2 beads (Merck Millipore). The immunoprecipitates were eluted with RIPA buffer containing the 100 ug/mL 3xFLAG peptide and subjected to immunoblot analysis.

### 4.8. RNA Interference for MerTK and STK38

Annealed oligonucleotides corresponding to short hairpin RNAs (shRNAs) against MerTK and STK38 were ligated into the BglII and HindIII restriction sites of the pSUPER-retro-puro retroviral plasmid (OligoEngine, Seattle, WA, USA). The sequences of oligonucleotides used to construct shRNA retroviral constructs were as follows: sh-mMerTK (#1: 5′-GATCCCCGGTGAAAGCTGTCAAGTCATTCAAGAGATGACTTGACAGCTTTCACCTTTTTA-3′ and 5′-AGCTTAAAAAGGTGAAAGCTGTCAAGTCATCTCTTGAATGACTTGACAGCTTTCACCGGG-3′; #2: 5′-GATCCCCGGGAAGAGACCGAGCTAGATTCAAGAGATCTAGCTCGGTCTCTTCCCTTTTTA-3′ and 5′-AGCTTAAAAAGGGAAGAGACCGAGCTAGATCTCTTGAATCTAGCTCGGTCTCTTCCCGGG-3′), sh-hMerTK (#1: 5′-GATCCCCGGTCTGTAATGGAAGGAAATTCAAGAGATTTCCTTCCATTACAGACCTTTTTA-3′ and 5′-AGCTTAAAAAGGTCTGTAATGGAAGGAAATCTCTTGAATTTCCTTCCATTACAGACCGGG-3′; #2: 5′-GATCCCCACTAGAAGATGTTGTGATTATTCAAGAGAAATCACAACATCTTCTAGTTTTTTA-3′ and 5′-AGCTTAAAAAACTAGAAGATGTTGTGATTTCTCTTGAAAATCACAACATCTTCTAGTGGG-3′), sh-mSTK38 (#1: 5′-GATCCCCGGACAAAGACCCTGATCTATTCAAGAGATAGATCAGGGTCTTTGTCCTTTTTA-3′ and 5′-AGCTTAAAAAGGACAAAGACCCTGATCTATCTCTTGAATAGATCAGGGTCTTTGTCCGGG-3′; #2: 5′-GATCCCCGAGAATAAGAATAGTGGTATTCAAGAGATACCACTATTCTTATTCTCTTTTTA-3′ and 5′-AGCTTAAAAAGAGAATAAGAATAGTGGTATCTCTTGAATACCACTATTCTTATTCTCGGG-3′), sh-hSTK38 (#1: 5′-GATCCCCGAGCAGAGTTCTTTGTATATTCAAGAGATATACAAAGAACTCTGCTCTTTTTA-3′ and 5′-AGCTTAAAAAGAGCAGAGTTCTTTGTATATCTCTTGAATATACAAAGAACTCTGCTCGGG-3′; #2: 5′-GATCCCCCGTCGGCCATAAACAGCTATTCAAGAGATAGCTGTTTATGGCCGACG TTTTTA-3′ and 5′-AGCTTAAAAACGTCGGCCATAAACAGCTA TCTCTTGAA TAGCTGTTTATGGCCGACG GGG-3′).

### 4.9. Immunofluorescence Analysis

NIH-3T3 cells infected with a mouse FLAG-MerTK, HA-STK38, Ras (G12V) expression retrovirus were fixed with 4% formaldehyde in PBS for 20 min. Then, cells were permeabilized with 0.1% Triton X-100 in PBS for 15 min and sequentially stained with anti-FLAG (Merck Millipore, F3165), Alexa Fluor 488 (Thermo Fisher Scientific, A11001) or anti-HA (Cell Signaling Technology, 3724), Alexa Fluor 594 (Thermo Fisher Scientific, A11072). The nucleus was visualized by the addition of DAPI. For F-actin staining, HEK293T cells were fixed with 4% formaldehyde in PBS for 20 min followed by permeabilizing with 0.1% Triton X-100 in PBS for 15 min. After the permeabilization, cells were incubated with Phalloidin-iFlour 594 regent (Abcam, ab176757, Cambridge, MA, USA) for 60 min. The nucleus was visualized by the addition of DAPI (Dojindo Laboratories, Kumamoto, Japan). Images were captured from each surface using a Keyence BZ-X710 microscope with Keyence software (BZ-X Viewer, version 01.03.02.01) (Keyence Corporation, Osaka, Japan).

### 4.10. Migration Assay

A total of 5 × 10^4^ NIH-3T3 cells were seeded into the upper chambers of transwell inserts (24-well, 8-µm pore size with a polycarbonate membrane; Corning Costar, Lowell, MA, USA) containing 500 µL of serum-free medium. The lower inserts were filled with 1 mL complete medium. Inserts were incubated at 37 °C for 5 h. At the end of the incubation, non-migrating cells in the upper surface of the membrane were removed with a cotton swab. Migrated cells in the lower inserts were fixed with methanol, stained with Giemsa (Nacalai Tesque), and counted using a light microscope (FSX100; Olympus, Tokyo, Japan).

### 4.11. Measurement of Cell Proliferation and Cell Viability

Cells were seeded in 35 mm dishes at 2  ×  10^4^ cells/dish and cultured at 37 °C in 5% CO_2_ incubator. To obtain the cell growth curve, the number of cells in each dish was counted 2 and 5 days after seeding. Triplicate dishes were prepared and counted for each cell type at every time point. For assessment of cell viability, cells were harvested and stained with Trypan Blue vital dye (Merck Millipore) 2 and 5 days post-seeding. Viable and non-viable cells were counted using a Countess automated cell counter (Thermo Fisher Scientific). For each cell type and time point, three dishes were seeded and counted.

### 4.12. In Vitro Pulldown Assay

To analyze Rac and Cdc42 activity, HEK293T cells were infected with retroviruses harboring MerTK shRNA (shMerTK #1 and #2), STK38 shRNA (shSTK38 #1 and #2), or luciferase shRNA as a control shRNA (shLuc). Cells were washed once with PBS and lysed in buffer P (20 mM Tris-HCl pH7.5, 150 mM NaCl, 10 mM MgCl_2_, 0.1% NP-40, 50 μM GTP, and a protease inhibitor and phosphatase inhibitor [Nacalai Tesque]). Cell lysates were incubated with GST-CRIB and glutathione-Sepharose at 4 °C for 1h. The resins were washed three times with buffer P and boiled in the Laemmli sample buffer. Rac1 and Cdc42 in the input and eluted samples were analyzed by immunoblotting.

### 4.13. Statistical Analysis

Each experiment was repeated multiple times, and quantitative data were obtained from at least three independent replicates; the data are expressed as the mean ± standard deviation. Significant differences between two groups were analyzed using an unpaired two-tailed Student’s *t*-test.

## 5. Conclusions

Our study highlights the critical role of STK38 in oncogenic Ras-induced cell migration. STK38 enhances MerTK tyrosine phosphorylation and oncogenic Ras-induced cell migration in a kinase activity-dependent manner. MerTK and STK38 knockdown reduced the activities of Rac1 and Cdc42, which are key regulators of cell migration. These findings provide a strong rationale for further research into MerTK and STK38 as potential therapeutic targets for the treatment of Ras-driven cancers.

## Data Availability

The data supporting the present results are available from the corresponding author upon request.

## Supplementary Materials

The following supporting information can be downloaded at https://www.mdpi.com/article/10.3390/ijms262110388/s1.
