# Supplementary material for: STK38 Kinase Promotes Cell Migration Induced by Oncogenic Ras via MerTK Activation
Source: Int J Mol Sci. 2025 Oct 25;26(21):10388. doi: 10.3390/ijms262110388 (PMC12607517; doi:10.3390/ijms262110388)
Supplement: Supplementary file 1 [file ijms-26-10388-s001.zip › ijms-3765023-supplementary.pdf]

MerTK-interacting proteins

Protein Name	Accession No.	Calculated MW/pI	Peptide Matched	Sequence Covered %	MASCOT Score
<u>Signal transduction-related proteins</u>					
Rho-related BTB domain-containing protein 1 (RhoBTB1)	Q9DAK3	78.8/6.5	1	1.6	26.5
Rho GTPase-activating protein 18 (ARHGAP18)	Q8K0Q5	25.6/8.4	1	7.2	21.2
serine/threonine-protein kinase 38 (STK38)	Q91VJ4	54.1/6.9	4	12.9	195.3
<u>Actin cytoskeleton</u>					
subunit of Arp2/3 complex subunit 2 (ARPC2)	Q9CVB6	34.3/6.8	2	7	90.8
subunit of Arp2/3 complex subunit 3 (ARPC3)	Q9JM76	47.3/5.6	1	2.4	32.8
Tropomodulin-3 (TMOD3)	Q9JHJ0	39.5/5.0	2	8.8	123.6
ALX homeobox protein 1 (ALX1)	Q8C8B0	36.9/8.8	1	2.5	23.8
<u>Others</u>					
serine beta-lactamase-like protein (LACTB)	Q9EP89	60.7/9.0	1	2	30.5
DnaJ homolog subfamily B member 9 (DNJB9)	Q9QYI6	25.6/8.4	1	7.2	21.2
